# Supplementary material for: See-Star: a versatile hydrogel-based protocol for clearing large, opaque and calcified marine invertebrates
Source: EvoDevo. 2024 Jun 25;15:8. doi: 10.1186/s13227-024-00228-0 (PMC11201320; doi:10.1186/s13227-024-00228-0)
Supplement: Supplementary file 1 — Additional file 1. Figure S1. Normalized sample depth of cleared P. miniata juveniles. Quantification of average sample intensity over normalized sample depth for the different clearing methods assayed on P. miniata juveniles. n = 5 per condition. Figure S2. High-resolution images of selected cleared samples used for IHC (A-H) and HCR FISH (I-J). A 2-TF Patiria miniata juvenile from Fig. 3A. B 7-TF P. miniata juvenile from Fig. 3B. C 11-TF P. miniata juvenile from Fig. 3C. D 11-TF P. miniata juvenile from Fig. 3F. E 16-TF Henricia sp. juvenile from Fig. 3I. F 11-TF Strongylocentrotus purpuratus. juvenile from Fig. 3L’. G Dorytheuthis opalescens paralarva from Fig. 3O. H Lepidozona sp. juvenile from Fig. 3Q. I P. miniata juvenile from Fig. 4C. J P. miniata juvenile from Fig. 4F. Figure S3. Papulae in Patiria miniata and Henricia sp. A Brightfield image of the aboral surface of an arm in a live P. miniata juvenile, showing the protruding papulae. B-E Acetylated α-tubulin IHC showing the aboral surface of a 11-TF P. miniata juvenile (B, D) and a 16-TF Henricia sp. juvenile (C, E). D, E Magnifications of the regions outlined in B and C respectively, showing details of the papulae. m: madreporite; pa: papula. Figure S4. See-Star clearing of Doryteuthis opalescens and Lepidozona sp. Brightfield images of representative cleared D. opalescens paralarvae (A) and Lepidozona sp. (B) imaged before clearing (following fixation) and after clearing. Squares = 600 µm. [file 13227_2024_228_MOESM1_ESM.docx]

# Supplementary information

**See-star protocol**

1. Let the specimens clear stomach contents for at least a couple of hours and ideally a couple days in clean filtered sea water (FSW), as microalgae are autofluorescent, and this signal can be maintained through the clearing process.
2. Anesthetize the specimens by incubating them in a 1:1 mix of FSW and 7.5% MgCl until they are completely relaxed.
3. **Fixation step:**

**Note:** The fixation solution contains paraformaldehyde, which is a toxic chemical. The fixation step and subsequent washes should be performed in a fume hood with appropriate personal protection equipment and waste management.

1. Remove as much FSW as possible and incubate the samples in fixation solution.
2. For small samples (for instance embryos, larvae, or samples < 1 mm), fix for 2-4 hours at room temperature, with gentle rocking.
3. For medium samples (for instance squid paralarvae, small echinoderms juveniles, or samples < 5 mm^2^), fix overnight at 4°C, with gentle rocking.
4. For large samples (for instance large echinoderm juveniles, small chitons and bivalves, or samples < 10 mm^2^), fix for 24-48 hours at 4°C with gentle rocking. Not that larger samples are limited by mounting and imaging practicality for most microscopy set-ups.

**Note:** The goal of this step is to allow for complete fixation and cross-linking between the acrylamide monomers and the proteins and nucleic acids within your sample. Thus, over fixation is not an issue; when in doubt about how long to fix, prefer a longer fixation time.

1. Rinse three times in 1X phosphate buffer saline (PBS) quickly, and then wash three times 20 minutes in 1X PBS
2. **Gelation step:**
3. Move the samples into a container that can be de-gassed (septum-capped vials, or septum-capped falcon tubes. Septa caps can be reused multiple times).
4. Remove as much PBS as possible and cover the samples using an excess of gelation solution, they should have at least ~1 cm excess solution above them.
5. Acrylamide polymerization is inhibited by oxygen; degassing helps to mitigate this effect. De-gass the samples before the overnight incubation at 4°C:
6. Using a 22G syringe connected to an active vacuum line, pierce the septum cap to draw out the air in the container. Avoid sticking the syringe into the liquid. Gently tapping the vial on the edge of a bench helps release bubbles, and inserting and removing the syringe a few times can help maximize the amount of air removed.
7. The process is complete when there are no apparent bubbles in the sample vial.
8. If degassing is not feasible (e.g., if you don’t have septum-capped vials or no vacuum) you can still successfully gel your solution if you include excess gelation solution; the top several millimeters won’t gel, but everything below still should.

4. Incubate overnight at 4°C.

5. After overnight incubation, de-gass the sample vials again using the same procedure.

6. Incubate the samples at 37°C for 2 hours. Gelation is complete when the solution becomes viscous; a clear boundary should be barely visible between the polymerized solution around your sample and the layer of water or unpolymerized solution at the surface.

1. **Decalcification step:**
2. Rinse samples 3 times with PBS to remove gelation solution, then add decalcification solution.
3. Incubate the samples in decalcification solution at 37°C until they are completely decalcified. For large or highly calcified samples, this step can last several days. The decalcification solution should be changed daily. Increasing the temperature to 55°C speeds up the process, but can be damaging for fragile samples.
4. Check the samples regularly. Loss of refraction from the calcium crystals in shells or skeletal structures indicate that the process is complete.
5. **Delipidation step:**
6. Quickly wash the samples with clearing solution.
7. Incubate the samples in clearing solution at 37°C. As for decalcification, the speed of the process can be increased by incubating at 55°C. Gentle rocking is recommended if the samples are not fragile.
8. Check the samples regularly. Partial transparency and translucency in the tisses indicate that the process is complete.

**Note:** the length, temperature and rocking of the clearing step have to be determined empirically by trial and error for different tissues and species. Prepare extra samples and plan to lose a few in the process; if you only have one precious sample, the safest condition is at 37°C with no shaking. Clearing is most rapid at 55°C with shaking, but at higher temperatures tissue warping and degradation are sometimes an issue.

1. Thoroughly wash the samples into 1X PBS.
2. **Staining step:**

From that point, proceed to downstream applications (immunohistochemistry or *in situ* hybridization).

**Note:** a few modifications to your downstream application protocols can be made, depending on the type of samples:

- For samples larger than 500 µm, at least double the length of your primary and secondary antibody incubations, and also increase the length and number of washes.
- Do not use phalloidin staining, actin has been denatured.
- Expect to troubleshoot antibodies: some antibodies don’t work as well in cleared tissues as they do with a standard PFA fixation (presumably due to denaturation of the tertiary structure of the epitope)

1. Following immunohistochemistry or in situ hybridization, move the samples in to CUBIC-mount (or another refractive index matching solution like glycerol).
2. Incubate overnight at 4°C in CUBIC-mount.
3. Wash the samples with a fresh volume of CUBIC-mount.
4. Mount your samples as desired for imaging.

**Note:** coverslip-bottom dishes are very useful for larger samples

Fixation solution:

- 4% paraformaldehyde
- 30% Acrylamide
- 3.3X PBS (this corresponds to the salinity of seawater)

Gelation solution

- 4% weight/volume acrylamide
- 0.25% weight/volume VA-044 (gelation agent)
- 1X PBS

Prepare using ice-cold reagents to avoid triggering the gelation process.

**Note:** excess Gelation Solution can be stored at 4°C for 1-2 days, or frozen at -20ºC for several months. To check the stability of stored solutions, add 25 µL of Bis-Acrylamide to 975 µL of hydrogel solution (to a final concentration of 0.05%) and heat at 37°C for 2 hours; this solution should polymerize into a soft gel.

Decalcification solution:

- 200 mM NaCl
- 50 mM Tris
- 500 mM EDTA pH 8.5

Adjust to pH 8.5 with NaOH.

Clearing solution:

- 300mM SDS
- 10mM Boric Acid
- 100mM Sodium Sulfite

Adjust to pH 9.0 with NaOH.

Mounting solution

- 50% weight/volume sucrose
- 25% weight/volume urea
- 25% weight/volume quadrol (N,N,N′,N′-Tetrakis(2-hydroxypropyl)ethylenediamine)

# Supplementary figures


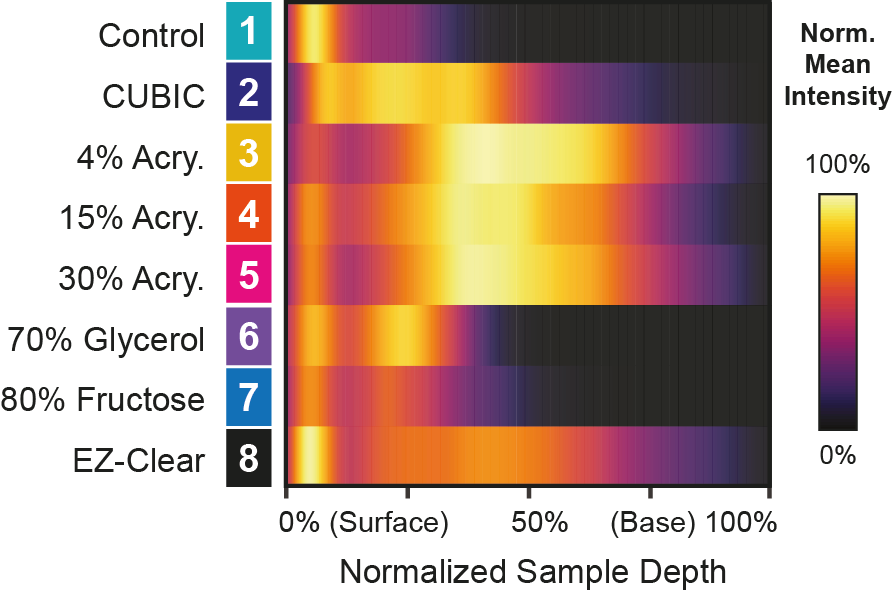


## Figure S1: Normalized sample depth of cleared *P. miniata* juveniles

Quantification of average sample intensity over normalized sample depth for the different clearing methods assayed on *P. miniata* juveniles. n = 5 per condition.

##
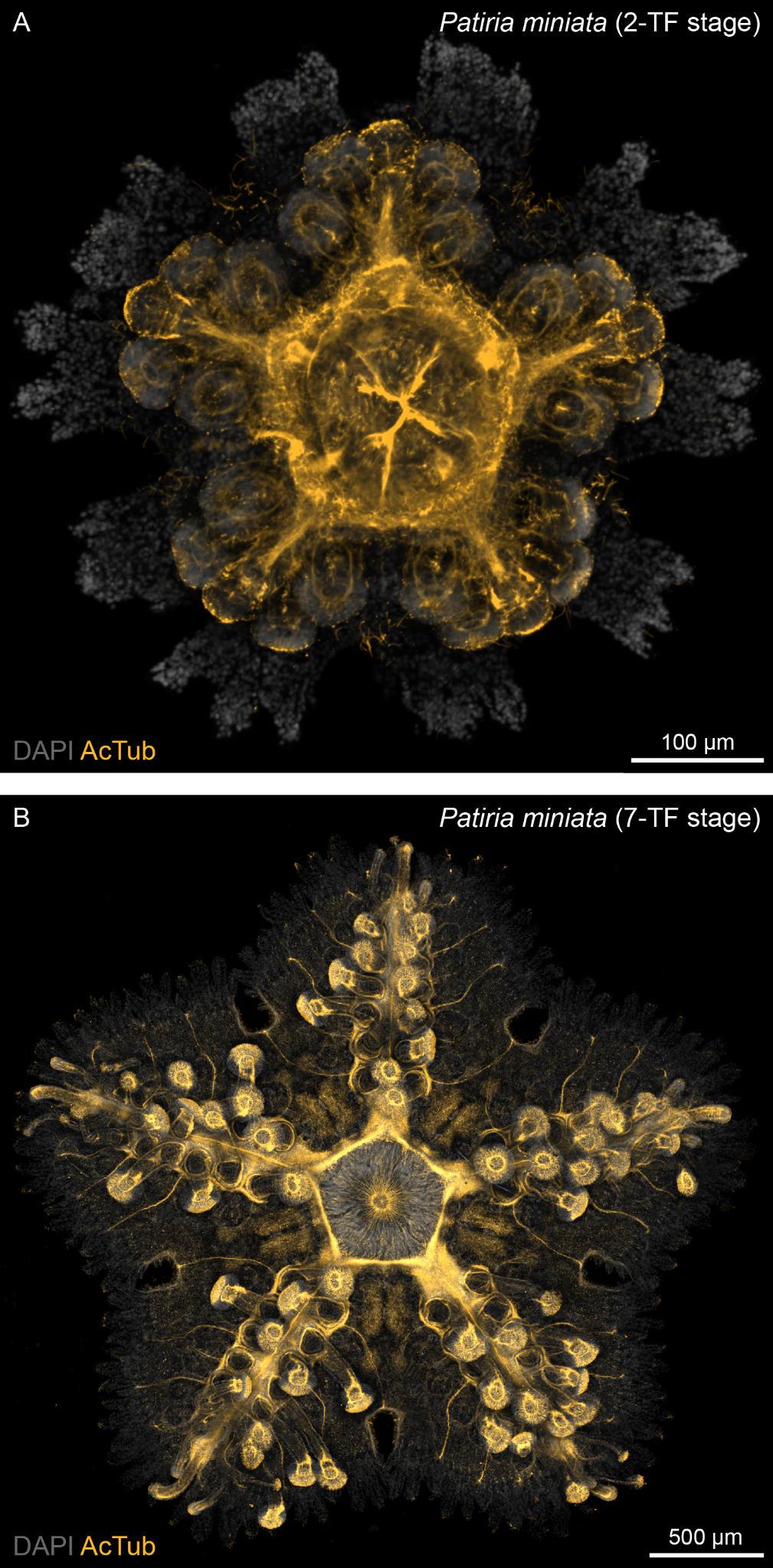


##

## Figure S2: High-resolution images of selected cleared samples (1/5).


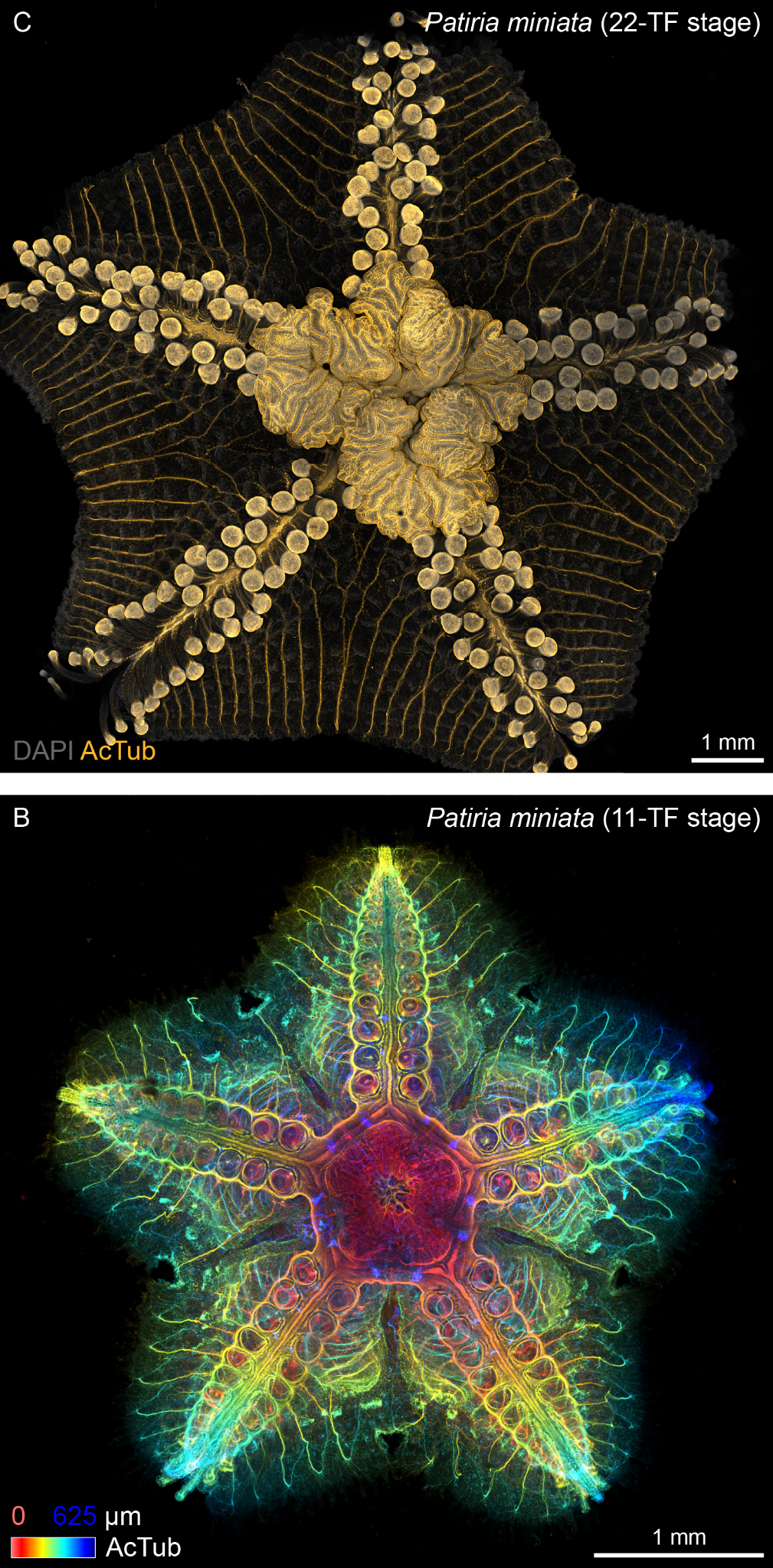


Figure S2: High-resolution images of selected cleared samples (2/5).


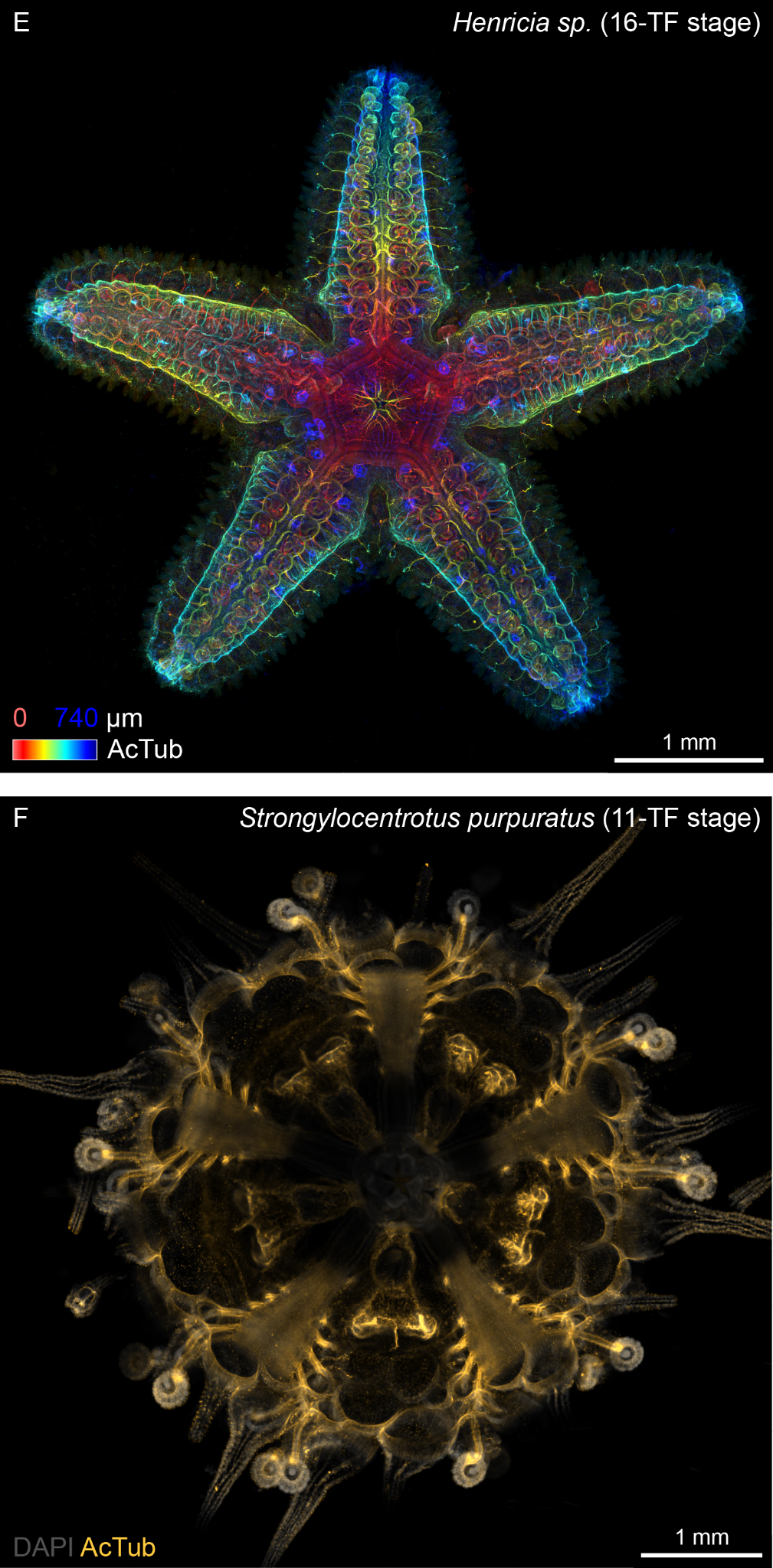


Figure S2: High-resolution images of selected cleared samples (3/5).


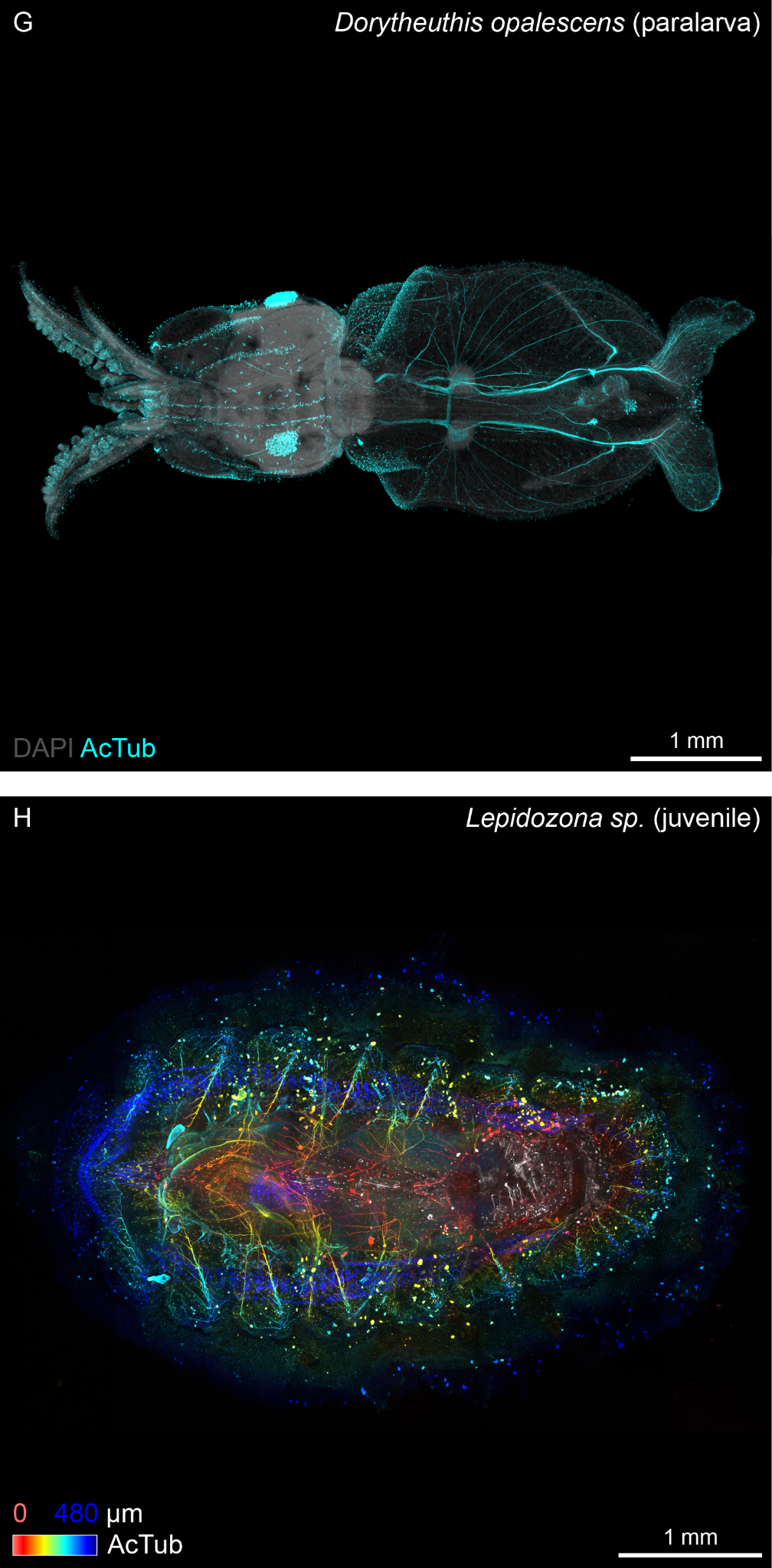


Figure S2: High-resolution images of selected cleared samples (4/5).


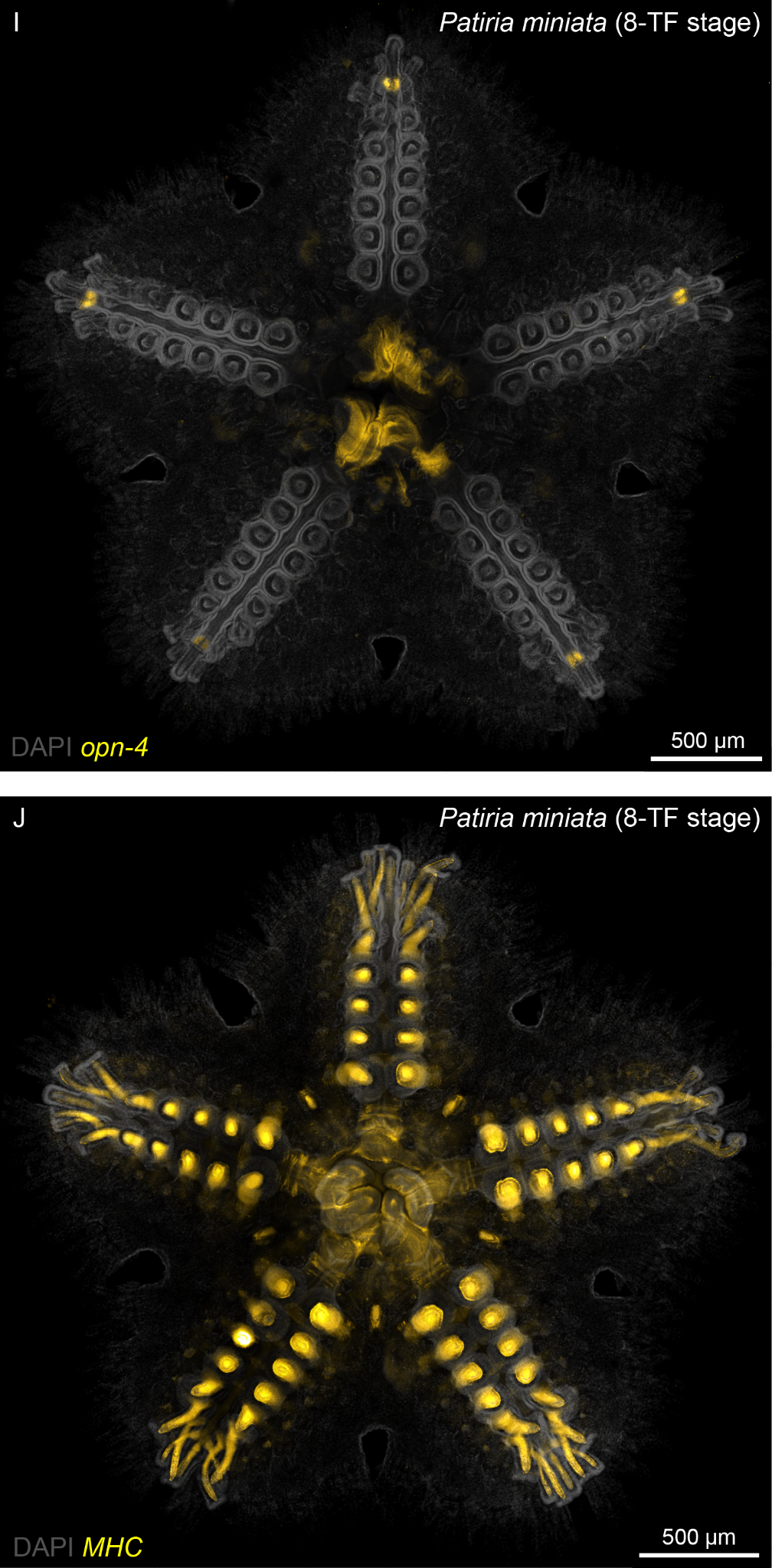


Figure S2: High-resolution images of selected cleared samples (5/5).

High-resolution images of samples used for immunohistochemistry (**A-H**) and HCR (**I-J**). **(A)** 2-TF *Patiria miniata* juvenile from Fig. 3A. **(B)** 7-TF *P. miniata* juvenile from Fig. 3B. **(C)** 11-TF *P. miniata* juvenile from Fig. 3C. **(D)** 11-TF *P. miniata* juvenile from Fig. 3F. **(E)** 16-TF *Henricia sp.* juvenile from Fig. 3I. **(F)** 11-TF *Strongylocentrotus purpuratus.* juvenile from Fig. 3L’. **(G)** *Dorytheuthis opalescens* paralarva from Fig. 3O. **(H)** *Lepidozona sp.* juvenile from Fig. 3Q. **(I)** *P. miniata* juvenile from Fig. 4C. **(J)** *P. miniata* juvenile from Fig. 4F.


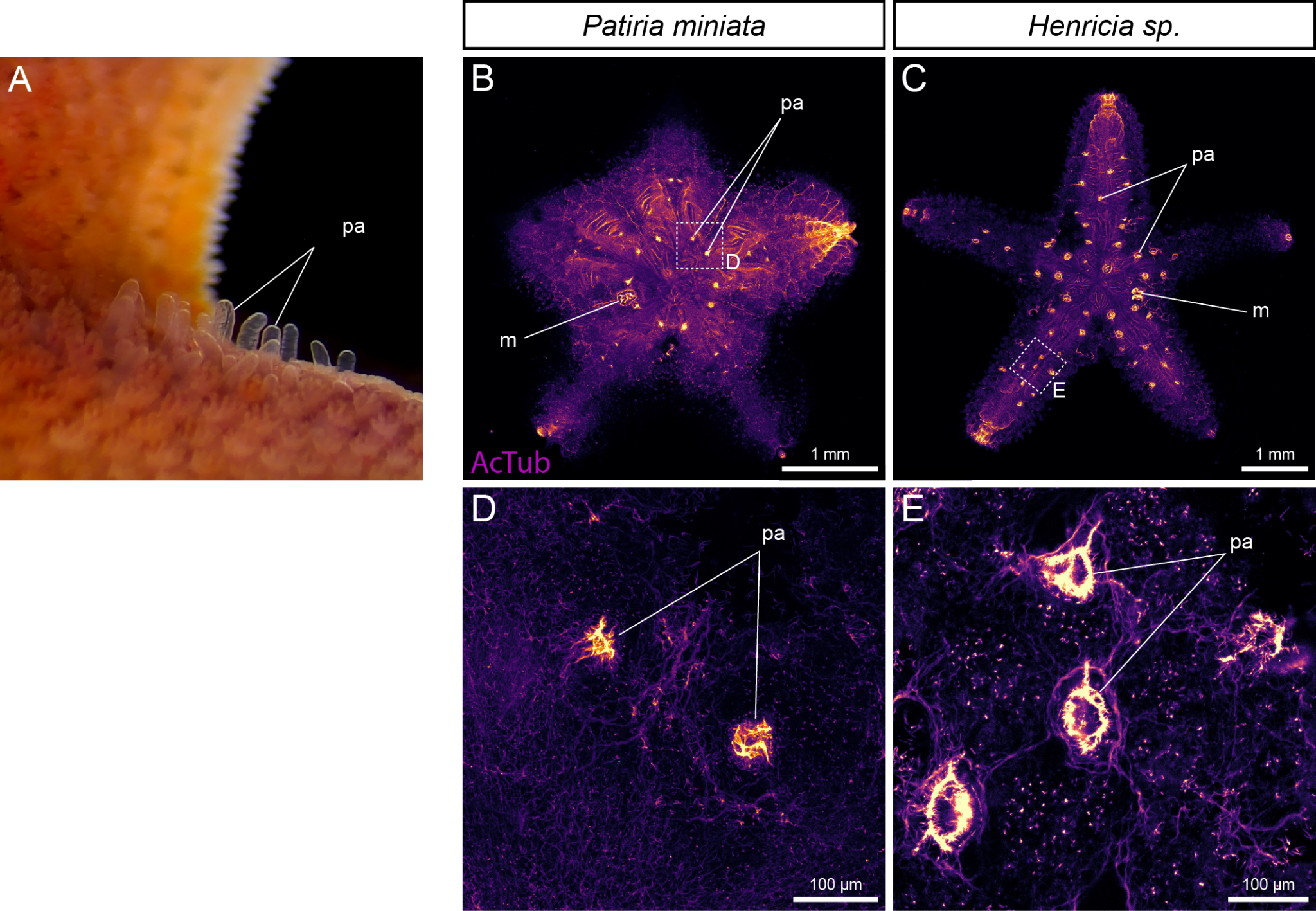


##

## Figure S3: Papulae in *Patiria miniata* and *Henricia sp*.

**A,** Brightfield image of the aboral surface of an arm in a live *P. miniata* juvenile, showing the protruding papulae. **B-E,** Acetylated α-tubulin IHC showing the aboral surface of a 11-TF *P. miniata* juvenile (**B, D**) and a 16-TF *Henricia sp*. juvenile (**C, E**). **D, E,** Magnifications of the regions outlined in **B** and **C**, respectively, showing details of the papulae. m: madreporite; pa: papula.


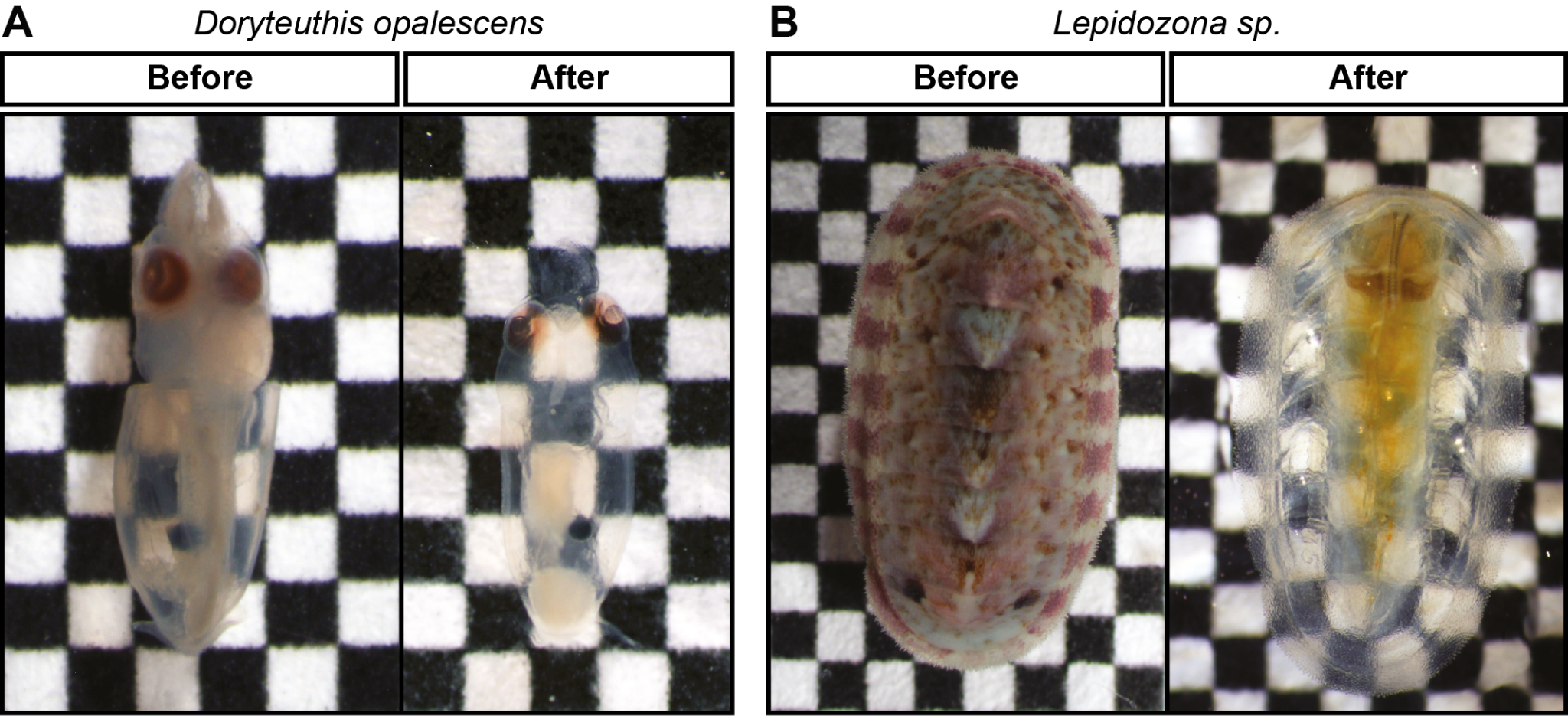


##

## Figure S4: See-Star clearing of *Doryteuthis opalescens* and *Lepidozona sp.*

Brightfield images of representative cleared *D. opalescens* paralarvae (**A**) and *Lepidozona sp.* (**B**) imaged before clearing (following fixation) and after clearing (before imaging). Squares = 600 µm.
